# Meeting of the Central Hospital Council

**Published:** 1911-06-24

**Authors:** 


### MEETING OF THE CENTRAL HOSPITAL COUNCIL.

The Central Hospital Council for London held a meeting on Thursday last week at St. Thomas's Hospital, when the following resolution was passed :

"That the Central Hospital Council for London, having regard to the effect which the National Insurance Bill must have upon the work of the voluntary hospitals all over the country, desires to impress upon his Majesty's Government the necessity of sufficient time being allowed for the consideration of the Bill in all its aspects by those who are now and have been in the past responsible for the medical relief of a large section of the people."

Mr. William West, Chairman of the Council, has written to the papers pointing out that "it seems difficult to understand in a Bill which must so greatly affect the position of the voluntary hospitals that those who for so long have been responsible for the medical relief of a large section of the community and in a large measure for the medical teaching in the medical schools should not have been consulted and that the question of the future of the voluntary hospitals should have been ignored."

